# Disease Ontology: improving and unifying disease annotations across species

**DOI:** 10.1242/dmm.032839

**Published:** 2018-03-01

**Authors:** Susan M. Bello, Mary Shimoyama, Elvira Mitraka, Stanley J. F. Laulederkind, Cynthia L. Smith, Janan T. Eppig, Lynn M. Schriml

**Affiliations:** 1 The Jackson Laboratory, Bar Harbor, ME, USA; 2 Department of Biomedical Engineering, Medical College of Wisconsin, Milwaukee, WI, USA; 3 Department of Epidemiology and Public Health, Institute for Genome Sciences, University of Maryland School of Medicine, Baltimore, MD, USA

**Keywords:** Disease models, Mouse, Ontologies, Rat

## Abstract

Model organisms are vital to uncovering the mechanisms of human disease and developing new therapeutic tools. Researchers collecting and integrating relevant model organism and/or human data often apply disparate terminologies (vocabularies and ontologies), making comparisons and inferences difficult. A unified disease ontology is required that connects data annotated using diverse disease terminologies, and in which the terminology relationships are continuously maintained. The Mouse Genome Database (MGD, http://www.informatics.jax.org), Rat Genome Database (RGD, http://rgd.mcw.edu) and Disease Ontology (DO, http://www.disease-ontology.org) projects are collaborating to augment DO, aligning and incorporating disease terms used by MGD and RGD, and improving DO as a tool for unifying disease annotations across species. Coordinated assessment of MGD's and RGD's disease term annotations identified new terms that enhance DO's representation of human diseases. Expansion of DO term content and cross-references to clinical vocabularies (e.g. OMIM, ORDO, MeSH) has enriched the DO's domain coverage and utility for annotating many types of data generated from experimental and clinical investigations. The extension of anatomy-based DO classification structure of disease improves accessibility of terms and facilitates application of DO for computational research. A consistent representation of disease associations across data types from cellular to whole organism, generated from clinical and model organism studies, will promote the integration, mining and comparative analysis of these data. The coordinated enrichment of the DO and adoption of DO by MGD and RGD demonstrates DO's usability across human data, MGD, RGD and the rest of the model organism database community.

## Abstract (precis)

**Summary:** Analyzing diverse disease data requires a comprehensive, robust disease ontology to integrate annotations and retrieve accurate, interpretable results. MGD, RGD and DO are working in collaboration to achieve this goal.

## INTRODUCTION

Model organism data associated with human disease is a rapidly growing, valuable resource for clinicians and translational and basic science researchers, as well as bioinformaticians, seeking to discover new genetic disease associations and analyze individual or population disease susceptibilities. However, without the use of a common structured disease vocabulary, access to these data requires collecting data from various model organism databases and the scientific literature, collating the amassed information, and aligning the different terminologies used in each resource.

For example, to identify the mouse and rat models of Alzheimer disease, a researcher would need to collate information from dozens of Mouse Genome Database (MGD; Eppig et al., 2017) and Rat Genome Database (RGD; Hayman et al., 2016) webpages, application programming interfaces (APIs) or downloadable files. Then he/she would need to align the Online Mendelian Inheritance in Man (OMIM; Amberger and Hamosh, 2017) terms used in the MGD annotations with the corresponding terms from Rat Disease Ontology (RDO; Hayman et al., 2016) used in RGD annotations to determine when the mouse and rat were modeling the same subtype of Alzheimer disease. Further, he/she would need to determine whether the disease modeled corresponds to the one discussed in a reference tagged with a Medical Subject Headings (MeSH, https://www.ncbi.nlm.nih.gov/mesh) term or to the Orphanet (Rath et al., 2012) Rare Disease Ontology (ORDO) term associated with a particular gene. Such extensive collecting and aligning of different terminologies can significantly hamper studies and analyses and/or lead to conflicting conclusions if definitions among vocabularies are inconsistent. In addition, computational tools developed to take advantage of model organism data in a clinical setting must download and integrate data from multiple resources to have the greatest chance of analytical success (Hoehndorf et al., 2015; Orechia et al., 2015; Patterson et al., 2016; Vitali et al., 2016). Having a single disease ontology with cross-references to multiple disease vocabularies incorporating both etiology- and anatomy-based views will greatly simplify and enhance the efficiency of disease-related searches within and across species.

Multiple clinical disease vocabularies have been developed for specific cases of use [e.g. International Classification of Diseases (ICD): morbidity and mortality reporting, as described in Schriml and Mitraka, 2015]. However, these are not necessarily compatible with the data-collection needs for disease models or human clinical data. In the past, MGD and RGD struggled to find a comprehensive and appropriately structured, defined vocabulary for human disease annotations (Bello et al., 2012). Prior to the current work described here, MGD used OMIM and RGD used RDO (developed by RGD) for human disease annotations. The RDO includes terms from the Comparative Toxicogenomics Database's ‘MErged DIsease voCabulary’ [MEDIC; a combination of the MeSH ‘Diseases’ branch (branch ‘C’) and OMIM terms (Davis et al., 2012)] and an RGD-supplemented custom vocabulary of over 1600 terms. However, neither RDO nor OMIM were fully sufficient for the needs of disease annotation and disease data integration in MGD and RGD (Table 1). For MGD, many mouse models fall outside the scope of OMIM. Additionally, RGD's custom terms are not fully mapped to other disease vocabularies. Also, the structure of MeSH, inherent in the RDO vocabulary, does not support computational reasoning across the vocabulary (Fernandez-Llimos et al., 2017).

**Table 1.**
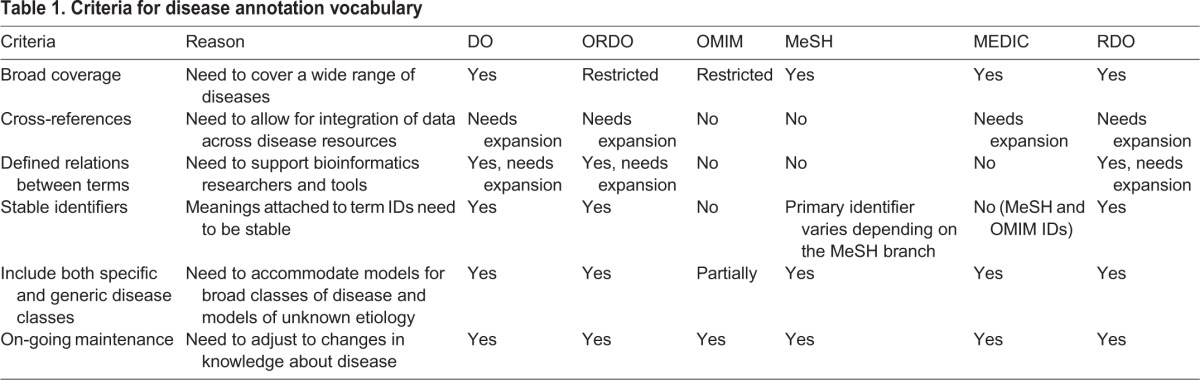
**Criteria for disease annotation vocabulary**

To be suitable for disease model annotation, a vocabulary needs to meet several criteria (Table 1). These criteria include: a sufficiently broad scope, cross-references for integration across disease resources, stability and maintenance of the vocabulary, a structure that supports reasoning over the vocabulary, permanent stable identifiers, and support for multiple types of users (see below). Several different vocabularies have been evaluated by MGD and RGD, including OMIM, MeSH, MEDIC, RDO, ORDO and Disease Ontology (DO; Schriml and Mitraka, 2015). Two major medical terminologies were not included in the evaluation, the ICD and the Systematized Nomenclature of Medicine (SNOMED) vocabularies. The ICD vocabularies, developed primarily for medical billing, contain many non-disease terms related to injuries and medical procedures that would require extensive filtering prior to use. The SNOMED vocabulary was excluded due to licensing issues for non-US users. Each of the evaluated vocabularies was developed for a specific purpose that was related to the annotation needs of MGD and RGD. OMIM strives to be the full catalog of human Mendelian phenotypic variation and includes extensive written histories of Mendelian diseases and their range of expression. ORDO is a structured vocabulary for rare diseases, capturing defined relationships between diseases, populated from literature and validated by international experts. In addition to disease records, both OMIM and Orphanet create mappings between their disease terms and the genes and genomic features linked to these diseases. MeSH is designed to index biomedical information in the National Library of Medicine's databases, including MEDLINE and PubMed. The DO's primary purpose is to provide the biomedical community with a consistent, reusable and sustainable resource for descriptions of all human diseases with extensive cross-mapping to other disease vocabularies. DO provides a unified etiology-based human disease classification, maintaining up-to-date disease nomenclature with extensive cross-references to National Cancer Institute (NCI) Thesaurus, OMIM, MeSH, ICD9CM, ICD10, ICD10CM, Orphanet, National Organization for Rare Disorders (NORD), SNOMED Clinical Terms (CT), Unified Medical Language System (UMLS), Experimental Factor Ontology (EFO) (Kibbe et al., 2015) and custom subsets (e.g. DO_FlyBase_slim, DO_rare_slim, DO_cancer_slim, DO_AGR_slim). DO utilizes OMIM as the authoritative resource for Mendelian genetic diseases.

### Criteria for use in disease model annotation

#### Scope

Both MGD and RGD capture a wide variety of data on human disease models in rodents. These diseases range from classical single gene Mendelian diseases to complex multi-genic diseases to disease susceptibility genes to diseases caused by environmental conditions, and include both rare and common diseases. Therefore, any disease ontology adopted for use by MGD or RGD needs to include in its scope the full range of disease types represented in rodent disease models.

Of the vocabularies reviewed – DO, MeSH, MEDIC and RDO – all have a broad scope that encompass the full range of disease types captured by MGD and RGD. OMIM and ORDO each have a restricted scope that does not encompass all of the types of diseases captured by MGD and RGD. OMIM excels at covering Mendelian diseases, providing a rich source of information for users and physicians, but does not systematically capture non-Mendelian or environmentally induced diseases. ORDO captures mainly rare diseases and has entries for only select common or environmentally induced diseases. For example, DO, MeSH, MEDIC and RDO all have a term for ‘Parkinson disease’, but OMIM and ORDO only have terms for specific subtypes of Parkinson disease, such as Parkinson disease, late-onset (OMIM:168600) or rare parkinsonian disorder (ORPHA:68402).

#### Cross-references

A major goal for both MGD and RGD is to provide easy translation between model organism data and human data. To make this translation, any disease vocabulary used needs to support the integration of disease data annotated using multiple different terminologies. The presence of vocabulary cross-references enables this translation and also enhances the ability of users to search using their preferred vocabulary term and find the related data. The cross-references in both DO and ORDO include all the disease resources used by MGD and RGD for annotations. However, neither includes all of the cross-reference identifiers used in both MGD and RGD annotations. For example, both DO and ORDO include cross-references to OMIM but neither includes all of the OMIM IDs used by MGD in their cross-references. Similarly, both include MeSH identifiers as cross-references but neither has all of the MeSH IDs used by RGD.

#### Defined relations between terms

To support computational users and bioinformatics tool development, disease model annotations need to be made to a vocabulary with robust defined relations between terms. Defined relationships between two ontology terms are captured using the Open Biological and Biomedical Ontology (OBO) Foundry's Relation Ontology (RO, Smith et al., 2005); this allows data annotated to a highly specific (child) term to be inferred to be associated with all of the less specific (parent) terms that are related to it. For example, all mouse and rat models annotated to ‘autosomal recessive early-onset Parkinson disease 6’ (DOID:0060369) can also be inferred to be models of the parent term ‘early-onset Parkinson disease’ (DOID:0060894) and its parent term ‘Parkinson's disease’ (DOID:14330).

DO, RDO and ORDO use defined RO relationships in their ontologies. The DO primarily uses the ‘is a’ relation to indicate that a disease is a subtype of a broader disease class. ORDO uses a variety of relations, including ‘is a’ (for subtypes) and ‘part of’ (for diseases that span multiple broader classes). The RDO has defined all relations for both MEDIC terms and supplemental terms as ‘is a’ relations. However, because the structure is inherited from MeSH, which does not have an ontological structure, this can result in some questionable assertions for RDO. For example, Genital Neoplasms, Male (RDO:0005657) has the parent term Urogenital Neoplasms (RDO:0005656), but Urogenital Neoplasms has ‘is a’ relations to both Male Urogenital Diseases (RDO:0005655) and Female Urogenital Diseases (RDO:00005654) (https://rgd.mcw.edu/rgdweb/ontology/view.html?acc_id=DOID:9007150). Reasoning over RDO would result in the transitive assertion that Genital Neoplasms, Male is a subtype of Female Urogenital Diseases. Although there are connections between terms, these connections do not use RO-based defined terms that support computational reasoning. In MeSH, the connection between terms is further complicated by the fact that connections are dependent upon the branch of the vocabulary you are viewing. OMIM classifies the phenotype descriptions as autosomal, X linked, Y linked or mitochondrial in a non-hierarchical vocabulary with related phenotypes grouped into phenotypic series [e.g. the Parkinson disease phenotypic series (OMIM:PS168600) includes 30 OMIM records].

#### Stable identifiers

To minimize the need to review and revise existing annotations and to enable tracking of nomenclature changes over time, the disease vocabulary should have a stable relationship between an identifier and the meaning of a term attached to that identifier. Although this is a staple of ontology development (Smith et al., 2007), it is not a requirement when building a vocabulary or encyclopedia of diseases, nor should it be assumed that all such resources are built in the same way. For example, OMIM retains identifiers but may update the content related to a specific identifier such that the new phenotype object attached to the identifier is significantly different from the old phenotype object. For example, OMIM:168600 was revised from ‘Parkinson Disease’ to ‘Parkinson Disease, Late-Onset’. It is important to assess all ID to term changes for any resource utilized for annotation to capture data updates.

#### Inclusion of both specific and generic disease classes

Disease vocabularies, such as DO, provide the structure to enable disease models to be associated with a generic (parent) disease class or to specific subtypes. This is critical for annotation because disease models are not always made for a specific subtype of a disease. For example, there are many mouse models of Parkinson disease that cannot be associated with a specific subtype of Parkinson disease as delineated in OMIM (27 of 78 models in MGD). These mouse models may be strains, complex genetic models or environmentally induced models. For each case, there is a need to associate the model with the disease term at the appropriate level of specificity, whether to the general term Parkinson Disease or to one of the specific Parkinson disease subtypes (e.g. Parkinson Disease 6).

DO, ORDO, MeSH and MEDIC all include hierarchies with generic diseases and broad disease classes. OMIM does not include generic disease types or broad disease classes. However, OMIM has recently added phenotypic series for some diseases (e.g. Parkinson disease) that collect members of the closely related disease families. These series have been extremely helpful throughout this project.

#### On-going content and nomenclature maintenance

As disease knowledge continues to expand and evolve, disease vocabularies need to remain up-to-date with current knowledge. Additionally, cross-references to disease vocabularies need to be updated as other resource vocabularies change. All vocabularies reviewed work to incorporate the latest changes in disease understanding.

Of the disease vocabularies reviewed, only DO fulfills all of the criteria described above. Importantly, DO has the broad scope needed to cover the different types of disease models captured by MGD and RGD, the defined structure needed to support computational users, and includes in its goals the maintenance of cross-references to many other disease resources (Schriml and Mitraka, 2015). Having concluded that DO was the best option for disease curation for both MGD and RGD, a collaboration was begun between MGD, RGD and DO to expand and enhance select areas of DO to improve coverage of existing MGD and RGD disease annotations in each database and improve discovery of diseases based on the needs of users of both databases. This work initially focused on increasing the coverage of cross-references (OMIM and MeSH) in use for MGD and RGD disease model annotations, expanding the types of relationships between disease terms to include ‘contributes to condition’ for OMIM susceptibility to a disease term, and increasing the number of anatomy-based ‘located in’ definitions for DO terms to improve accessibility of disease data using DO. Below are the results of the ongoing collaboration between MGD, RGD and DO to enhance and expand the DO.

## RESULTS

### Disease collaborative coordination efforts

To coordinate disease-term reviews and updates, the MGD and RGD teams identified and shared with the DO team the subset of their disease terms where the term identifier was not already cross-referenced to a Disease Ontology ID (DOID) at the onset of this project. The shared files (disease term, RDO ID, OMIM ID, suggested parent term mappings) enabled collaborative review and addition of terms and cross-references into DO, and continue to foster ongoing discussions between the teams and provide MGD and RGD with updated DO term mappings. The team reviewed and classified each term and determined which terms were within the scope of DO, with only a small set of terms identified to be symptoms, injuries or phenotypes. GitHub tickets for ongoing work were entered in the public DO issue tracker (https://github.com/DiseaseOntology/HumanDiseaseOntology/issues). The use of a public issue tracker allows the MGD, RGD and DO teams, and additional interested parties, to access and track progress (Table 2).

**Table 2.**
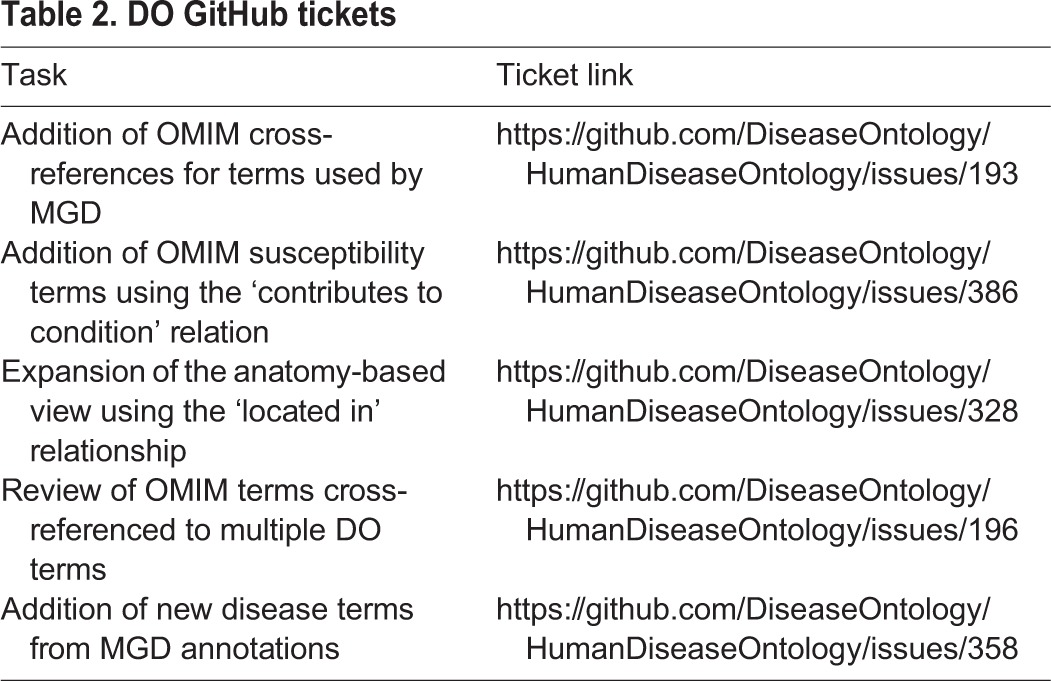
**DO GitHub tickets**

### Addition of cross-references

Disease identifiers in use by either MGD or RGD for disease annotation were compared to existing cross-references in DO to create a targeted set of potential new identifiers (Table 3) for addition to DO. The potential new cross-reference terms were reviewed to determine whether the disease term attached to the identifier (1) corresponded to an existing DO term, (2) represented a term within the scope of DO that should be added or (3) fell outside the scope of DO. Identifiers for disease terms that corresponded to existing DO terms were added as cross-references for the appropriate term. Terms that fell within the scope of DO but represented new diseases were collected for review and addition to DO. As part of the review, additional cross-references from other disease vocabularies were identified and added to DO.

**Table 3.**
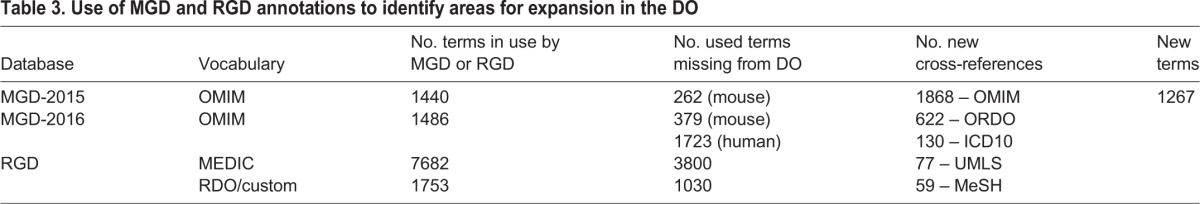
**Use of MGD and RGD annotations to identify areas for expansion in the DO**

Of the 1440 OMIM terms with mouse model annotations in MGD for the first round of review in December 2015, 1178 were already in DO, whereas 262 were identified for potential inclusion. This review was repeated in August 2016, at which point 379 OMIM terms were identified for review and potential inclusion in DO. This second review included terms from the 2015 review that had not yet been added to DO as well as new terms used in MGD annotations. In addition, the 2016 review identified OMIM terms with a human gene association that were not yet in DO. This second set contains terms also found on the list of terms with mouse models.

Of the 9435 RDO terms in use by RGD, 4605 were already in DO, whereas 4830 were reviewed for potential inclusion. RGD selected 565 terms for detailed review. To date, 164 of these have been reviewed and 48 were determined to be outside the scope of DO, whereas 116 were mapped to extant DO terms or used to create new DO terms. As part of the review process, additional terms closely related to the targeted term were identified; for example, if the target term is a member of an OMIM phenotypic series, then all members of the series would be reviewed and added to DO along with the targeted ID. Thus, the number of new cross-references often exceeds the number of targeted identifiers.

DO includes in its scope all human disease, but the reviews identified terms in use by MGD and RGD that fell outside this scope. These terms were reviewed and handled in different ways depending on the term. For MGD, there were two different sets of terms that fell outside the scope of DO. One set of these, the OMIM disease susceptibility terms, contributed to the expansion of relationships in DO (see below). The second set consisted of OMIM gene plus phenotype records that describe both a gene and one or more phenotypes or diseases associated with that gene. OMIM is actively working to eliminate this type of record – currently there are only 77 of these left out of 8000 OMIM phenotype entries (Ada Hamosh, Johns Hopkins University School of Medicine, personal communication) – and a review of MGD annotations is required to identify the diseases being modeled. For RGD, a significant number (>140) of terms were determined to be outside of DO's scope. These primarily related to injury (e.g. Acute Kidney Injury, RDO:0001702), infection (e.g. Cardiovascular Infections, RDO:0005485) or signs/symptoms (e.g. Diabetic Foot, RDO:0007073). RGD is continuing to use RDO to capture these annotations.

### Identification of novel disease classes

Both MGD and RGD need to incorporate disease mentions in publications that do not match an existing term in OMIM (MGD) or RDO (RGD). For MGD, these annotations are captured in the database in note fields and represent over 360 distinct diseases. These diseases are typically generic disease classes, such as heterotaxy, polycystic kidney disease or inflammatory bowel disease. RGD captures similar types of annotations by adding terms to RDO. RGD has added over 1700 terms to RDO to cover a range of diseases, from specific types of retinitis pigmentosa to acute pancreatitis (Hayman et al., 2016). Terms from both of these sets were compared with those in DO to determine whether they could be mapped to existing DO terms, represented diseases within the scope of DO that should be added, or fell outside the scope of DO (Table 3).

As part of the conversion from OMIM to DO at MGD, the alignments to DO created during this project were used to guide the annotation of the 600 mouse alleles with disease annotations in note fields. For MGD, this resulted in the addition of over 1070 new disease model annotations to DO disease terms for 330 different diseases. This review also identified 31 potential new DO disease terms that are in the process of being reviewed for addition to DO. In most cases, these new terms are more specific subtypes of existing DO disease classes.

### Refinement of OMIM cross-references

During the review and initial implementation of use of the DO at MGD and RGD, a need was identified to refine the OMIM cross-references in DO so that the mappings were made at a higher degree of granularity. OMIM IDs for multiple subtypes of a disease were frequently all associated with a generic term for the disease. This grouping of OMIM IDs complicates the differentiation of etiologically similar models from the full set of models. This grouping also complicates the translation of annotations made to OMIM terms by other resources. For example, the Human Phenotype Ontology (HPO) annotations (Köhler et al., 2017) are made directly to human disease terms from OMIM and ORDO. The grouping of multiple distinct OMIM or ORDO diseases into a single DO term results in HPO annotations for distinct subsets being merged into a common description, which may or may not accurately reflect the specific individual disease phenotypes. Thus, the decision was made to create individual entries for members of OMIM series whenever appropriate.

**Fig. 1.**
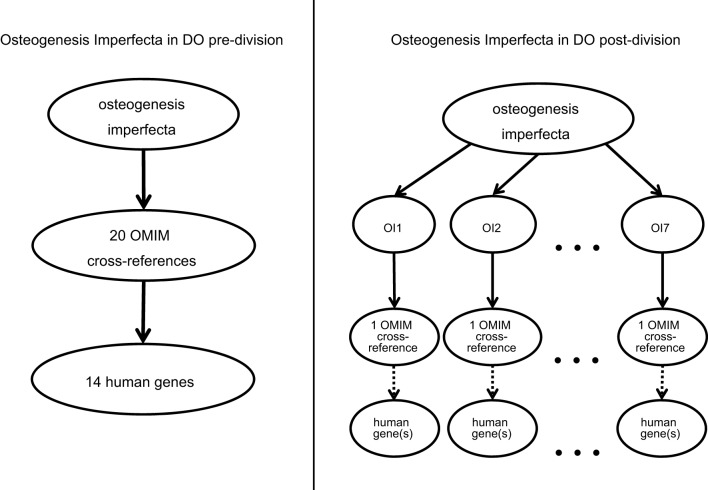
**Division of osteogenesis imperfecta (OI) terms in DO.** Increasing the granularity of OI terms in DO (shown on the right) allows for refined relationships of OMIM gene-to-disease associations (dotted arrows) to equivalent DO terms via OMIM cross-references to DO terms (solid arrows).

The initial review focused on 229 OMIM phenotypic series where annotations existed either in MGD or HPO (see Ticket link in Table 2). To date, 99 of these series have been reviewed, resulting in the addition of 815 new DO terms associated with over 1000 cross-references. For example, all of the OMIM cross-references for the OMIM phenotypic series ‘Osteogenesis imperfecta’ (OMIM:PS166200) had been associated with the DO term ‘osteogenesis imperfecta’ (DOID:12347). After review, individual DO terms were created for each member of the OMIM series and the appropriate cross-references (e.g. OMIM, ORDO and ICD10 IDs) added to the new DO term (Fig. 1). The OMIM phenotypic series ID was also entered as a cross-reference to the parent DO term. This process increased both the granular resolution of DO and refined the cross-reference relationships in DO.

### Expansion of DO relationship types

#### Addition of OMIM susceptibility terms

A number of MGD annotations had been made to OMIM susceptibility terms (e.g. ‘susceptibility to Alzheimer disease 9’, OMIM:608907; ‘susceptibility to malaria’, OMIM:611162). These terms represent susceptibility to the disease and not the disease itself, and are therefore outside the scope of DO. In these cases, adding the OMIM ID as a cross-reference to the DO term would be incorrect. However, creating a relationship between the OMIM ID and the DOID was desirable, as this allows the translation of associations between human genes and disease susceptibility in OMIM into associations between a human gene and DO term. Retaining access to this valuable set of gene annotations was deemed a high priority. To accomplish this, a new relationship was added to the DO, ‘contributes to condition’ (RO:0003304), and used to link OMIM susceptibility terms to DO terms. The ‘contributes to condition’ relationship is defined in RO as ‘A relationship between an entity (e.g. a genotype, genetic variation, chemical, or environmental exposure) and a condition (a phenotype or disease), where the entity has some contributing role that influences the condition’.

**Fig. 2.**
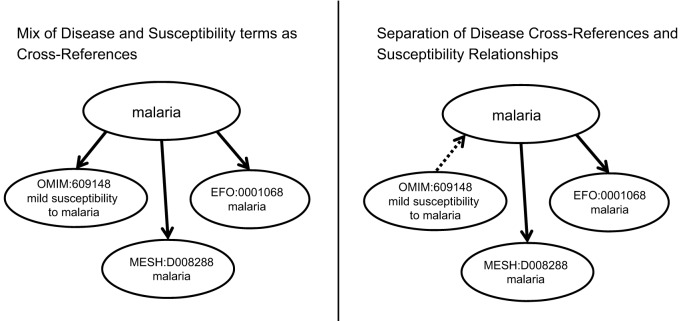
**Addition of ‘contributes to condition’ relations to DO.** Prior to this project, OMIM cross-references in DO contained a mix of primarily disease records plus a few ‘susceptibility to disease’ records (shown on the left). The addition of the new relation allows disease cross-references to be computationally distinguished from relationships between a disease and a susceptibility to that disease (shown on the right). Solid lines represent cross-references; dotted lines represent the ‘contributes to condition’ relationship.

A list of 181 OMIM susceptibility terms was created by searching the OMIM database. A review of this list identified 135 OMIM susceptibility terms that referred to a disease represented by a DO term. These terms were added to the DO file and connected to the related disease using the ‘contributes to condition’ relationship. For example, the OMIM term ‘susceptibility to malaria’ was related to the DO term for malaria (DOID:12365) via the ‘contributes to condition’ relationship (Fig. 2), allowing the OMIM gene information to be connected to the DO disease term.

The remaining 45 OMIM terms that do not reference an extant DO term fall into several classes. The largest class are terms that refer to susceptibility to infection by a type of bacteria or virus (e.g. susceptibility to herpes simplex encephalitis 1, OMIM:610551; susceptibility to mycobacterium tuberculosis, OMIM:607948). Others refer to susceptibility to develop disease-related complications (e.g. susceptibility to microvascular complications of diabetes 1, OMIM:603933) or to develop a phenotype (e.g. susceptibility to intracerebral hemorrhage, OMIM:614519). These terms will require further review to determine whether an appropriate mapping to a DO term can be created.

#### Enrichment of anatomy-based relations

**Fig. 3.**
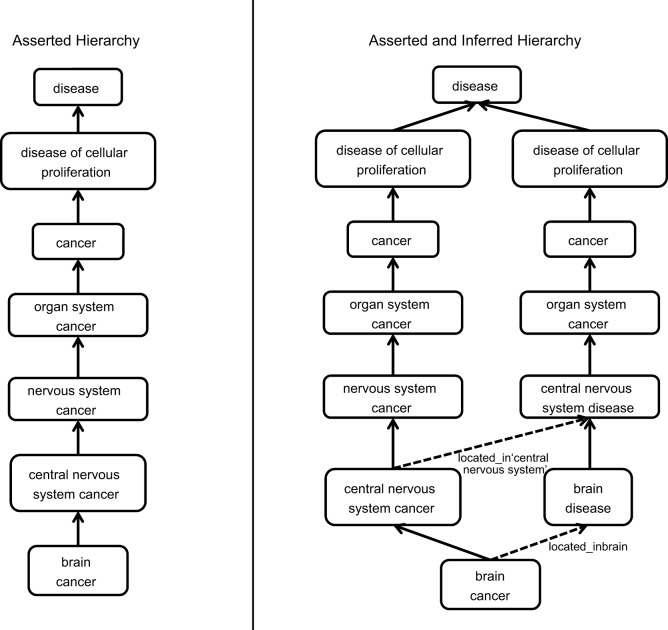
**Addition of ‘located in’ axioms and the derivation of additional ‘is a’ disease relationships.** Solid arrows indicate asserted relationships in DO; dashed lines indicate relationships inferred from anatomy-based axioms.

The DO uses the primary etiology of a disease to define the single asserted ‘is a’ relationship for each disease in DO (Schriml et al., 2012). However, not all users approach disease from the perspective of the primary cause(s). To meet the needs of users that approach disease from an anatomy-based perspective, an anatomical system-based review of DO was undertaken to identify terms where the addition of a ‘located in’ (RO:0001025) relationship to an anatomical term would enhance the usability of the DO for these users. For example, although, from an etiological perspective, cancers of the brain should be placed under cancer, a user with an anatomy-based perspective would expect to see this under brain disease. The addition of anatomy-based relations to a term allows for multiple parents to be inferred and displayed, thus meeting the needs of both sets of users. The cross-species anatomy ontology, UBERON (Mungall et al., 2012), is used for anatomy terms in these relationships as this ontology has been adopted by many other groups to facilitate cross-species comparisons. In the brain cancer example, the addition of a ‘located in’ relation to the UBERON anatomy term ‘brain’ (UBERON:0000955) and use of the UBERON structure allows us to infer the additional parent of ‘brain disease’. Thus, in MGD and RGD, ‘brain cancer’ is displayed under both the nervous system and cancer branches of the DO (Fig. 3). The review of diseases for addition of anatomy-based ‘located in’ information was made on a system basis. Initial reviews of the cardiovascular system disease and neurological disease branches have been completed. The DO now contains over 1000 ‘located in’ relationships. A breakdown of the spread of terms with these relationships in the branches of the DO is shown in Table 4. This total includes both the relationships added as part of the cardiovascular and neurological system reviews and ‘located in’ relationships that were added as part of other term reviews and new term additions.

**Table 4.**
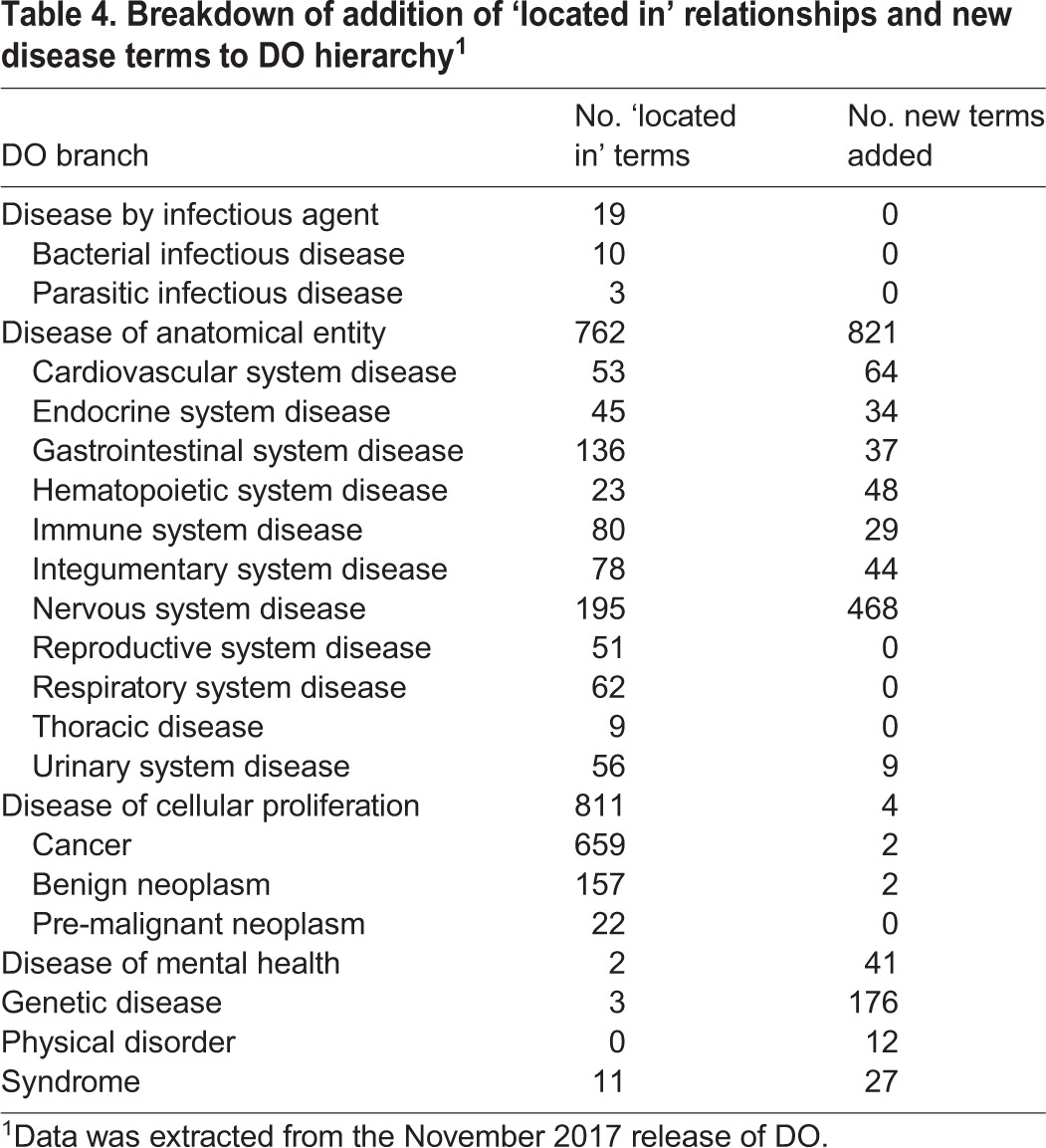
**Breakdown of addition of ‘located in’ relationships and new disease terms to DO hierarchy^1^**

### Identification of diseases in need of review

In some cases, placement of a disease term in the DO hierarchy is not straightforward. These are typically cases where different competing organization schemes of a disease series exist. These require more in-depth literature review and in some cases may require consultation with external medical experts. Classification of Parkinson disease illustrates this point, with over 20 subtypes defined in OMIM. Parkinson disease was classified into juvenile-, early- and late-onset based on the GeneReviews 2014 article, ‘Parkinson disease overview’ (Farlow et al., 2004). More granular DO terms corresponding to the OMIM subtypes were then assigned to the broader classes, unifying the classification approaches. In some cases the different vocabularies use classification schemes using different criteria. For example, Cockayne syndrome is classified by OMIM based on complementation groups (CSA, CSB) but in Orphanet based on phenotypic severity (CS1, CS2, CS3). OMIM initially had a record for CS3 (OMIM:216411) but, after discussions with OMIM leaders Ada Hamosh and Joanna Amberger, the OMIM record for CS3 was obsoleted as this had a single reference and does not correspond to the Orphanet record for CS3. Because of the competing classification approaches, the OMIM and Orphanet identifiers were not split out into subclasses. Experts will be consulted to clarify this disease.

### Refinement of existing OMIM cross-references

In a small set (53) of cases, the same OMIM ID was attached to more than 1 DO term. Those with MGD annotations (24 of 53) were reviewed to determine whether the duplicate placement was appropriate. In 17 of the 24 cases, the OMIM ID was removed from one of the DO terms. Most of these resulted from the OMIM record containing both a main disease entity (what the ID refers to) and an included disease entity (not what the ID refers to). For example, in OMIM, mast cell disease (OMIM:154800) includes the ‘included entity’ urticaria pigmentosa. As these are distinct diseases, using a single OMIM ID as a cross-reference for both would lead to incorrect inferences across the ontology. The remaining 8 cases required more extensive review and resulted in removal of the OMIM ID from both of the original DO terms.

### Overall expansion and enrichment of DO

As a result of this project, many new DO terms and cross-references have been added to DO. Over 1200 new DO terms were added. These include both terms for diseases that were missing from DO and terms that were created to represent subtypes of specific diseases. All new DO terms have a textual definition, and relevant cross-references from OMIM, ORDO, ICD10 and MeSH were added. The cross-references include over 1800 OMIM IDs, 620 ORDO IDs, 130 ICD10 IDs and 60 MeSH IDs. This covers approximately 90% of the terms currently used by MGD. To date, the collaborative review process also resulted in substantial updates to existing DO cross-references and the development of procedures for ongoing work on the DO. For example, MGD now supplies DO with weekly lists of new OMIM terms and changes to OMIM term meanings based on existing MGD quality control (QC) processes.

### Release of DO files

To meet the needs of different user groups, the DO produces different versions of the ontology. These include both OWL and OBO formatted versions of the file. Both simple (including only the asserted ‘is a’ etiology-based relationships) and merged (including both asserted and inferred relationships) are produced in each of the formats. New versions are produced following each approximately monthly release. These files can all be found on the DO GitHub site (https://github.com/DiseaseOntology/HumanDiseaseOntology). MGD and RGD both import the merged versions of the file that use the ‘located in’ and ‘contributes to’ relationships. For more details on the production and release of DO files, please see the DO GitHub site.

## DISCUSSION

The requirement for a robust disease ontology designed to support model organism disease annotations is shared by more groups than just MGD and RGD. The additions and enhancements made to DO as well as refinements made to the systems for requesting changes and additions make DO an excellent choice for model-based human disease annotations. Both MGD and RGD have converted or mapped, respectively, their systems to use the DO even while the work continues. In addition, the newly formed Alliance for Genome Resources (AGR, http://www.alliancegenome.org) has chosen to use DO for all disease annotations. Besides MGD and RGD, the AGR includes model organism databases for zebrafish (ZFIN), *Drosophila* (FlyBase), *Caenorhabditis elegans* (WormBase) and *Saccharomyces cerevisiae* (SGD). Implementation of use of the DO at MGD has already enhanced our ability to integrate data: annotations made by the HPO team to Orphanet diseases are now integrated in the Human-Mouse: Disease Connection tool (http://www.informatics.jax.org/humanDisease.shtml, Blake et al., 2017) at MGD. Prior to the DO implementation, MGD was unable to integrate these annotations with the existing OMIM annotations. A detailed description of the conversion/mapping to DO by MGD and RGD will be detailed in subsequent publications.

Expansion of the relationships in DO to include inferred ‘is a’ relationships based on ‘located in’ information has enhanced the accessibility of diseases based on different viewpoints. For example, benign renovascular hypertension (DOID:13145) had an asserted relationship to benign secondary hypertension (DOID:13143) but lacked a relationship to renal hypertension (DOID:1073). Thus, a user browsing kidney diseases would have missed data related to benign renovascular hypertension. The addition of a new relationship based on the information that this disease is located in the kidney allows for improved recall of the disease data for both manual and computational users.

The introduction of over 1200 new terms, 1900 cross-references and 1100 new relationships between terms has greatly enhanced the utility of DO for MGD, RGD and other model organism databases. A breakdown of where these terms fall within the DO hierarchy is shown in Table 4. The collaboration between MGD, RGD and DO is ongoing. Adoption of DO by this wide range of model organism databases will help to identify additional areas for expansion and enrichment. In addition, these changes will benefit the many tools and resources already using DO [e.g. Jax-CKB (Patterson et al., 2016); Triple-Negative Breast Cancer Database (Vitali et al., 2016), OncDRS (Orechia et al., 2015), QueryOR (Bertoldi et al., 2017), Disease Compass (Kozaki et al., 2017), DisSim (Cheng et al., 2016) and UniCarbKB (Campbell and Packer, 2016)] by increasing the amount of data these tools can import from other resources.

This collaborative effort has helped speed the integration of the diverse ecosystem of disease resources. The careful review of terms and refinement of cross-references will increase both the coverage of terms that can be retrieved as well as the accuracy of retrieved data. Having efficient, reliable and thorough integration of disease resources is a key element in bringing precision-medicine forward. Tools such as those for candidate gene analysis, tumor classification, drug repurposing and cancer variant analysis [e.g. CIViC (https://civic.genome.wustl.edu/home; Griffith et al., 2017)] require access to as much data as possible in order to provide researchers and clinicians with an accurate picture of our current understanding of disease. This project and the continued development of DO are essential in these efforts.

## MATERIALS AND METHODS

### Data collection

Scripts were run against the MGD and RGD databases on multiple occasions (12/2015 through 7/2017) to collect disease vocabulary terms in use in each database. Disease terms annotated to mouse or rat models with IDs not present in the DO file were output in files and shared using Google docs. Additional scripts were run against MGD to identify DO terms with multiple OMIM cross-references that also had mouse or HPO annotations. Annotation counts included in the outputs were used to prioritize more frequently used terms for review. OMIM terms cross-referenced to multiple DO terms and obsolete OMIM terms included in the DO file were identified using MGD QC reports during the implementation of the DO at MGD. Outputs from the scripts and QC reports can be accessed through issue tracker tickets on the DO GitHub site (Table 2). Data for Table 4 were extracted from the DO November 2017 release. MouseMine (Motenko et al., 2015) was used to derive the counts of terms for each DO branch.

### Term review and addition

The output of the scripts was manually reviewed to identify (1) OMIM IDs that were missing as cross-references in the DO (2) OMIM and RDO IDs that represented terms missing from the DO and (3) OMIM and RDO IDs representing terms that were out of scope for the DO. Multiple human disease resources (Table 5) as well as primary research articles were used during the review process. To facilitate new term addition, a standard definition template was developed and used whenever possible. This template has the form: ‘A [disease parent term] characterized by [distinguishing phenotype(s)] that has_material_basis_in [mutation type or zygosity] in [gene or genetic region] on [chromosome]’, where the square brackets were filled in with the relevant information. Term additions to the doid-edit.owl file were made either manually using Protégé 5.1 or by using the ROBOT tool (https://github.com/ontodev/robot). Terms were added using DOIDs rather than incorporating (MIREOTing) IDs from other vocabularies for several reasons, including: (1) DO is a domain ontology and thus uses DOIDs for terms, (2) ease of use of the ontology in curation, (3) avoiding incorporation of unstable identifiers and (4) integration of terms with the same meaning from multiple vocabularies into a single DO entry. Consistency of the ontology is checked using the ELK 0.4.3 reasoner. Members of the MGD, RGD and DO teams continue with their regular conference calls and periodic in-person meetings to discuss ongoing additions and modifications to DO and expansion of cross-references and term relationship representations. These calls facilitate a dynamic response to both changes in disease knowledge and the evolving needs of curation at MGD and RGD.

**Table 5.**
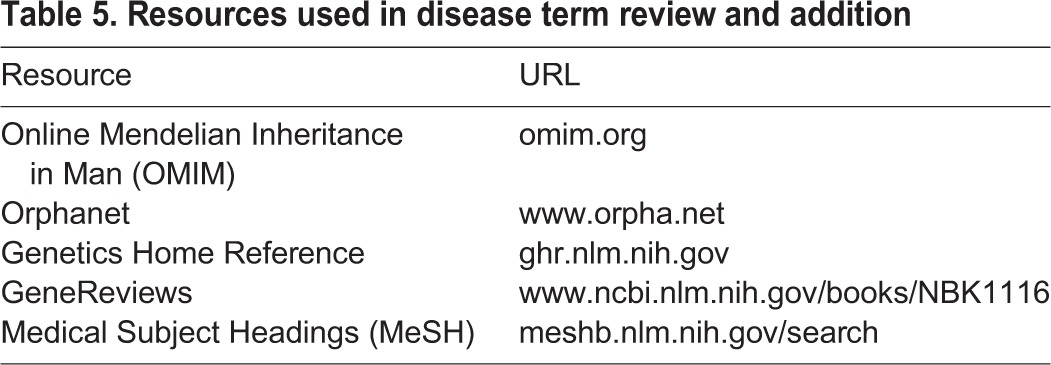
**Resources used in disease term review and addition**

## References

[DMM032839C1] Amberger J. S. and Hamosh A. (2017). Searching online mendelian inheritance in man (OMIM): A knowledgebase of human genes and genetic phenotypes. *Curr. Protoc. Bioinformatics*. 58, 1.2.1-1.2.12. 10.1002/cpbi.27 28654725 PMC5662200

[DMM032839C2] Bello S. M., Richardson J. E., Davis A. P., Wiegers T. C., Mattingly C. J., Dolan M. E., Smith C. L., Blake J. A. and Eppig J. T. (2012). Disease model curation improvements at Mouse Genome Informatics. *Database* 2012, bar063 10.1093/database/bar063 22434831 PMC3308153

[DMM032839C25] Bertoldi L., Forcato C., Vitulo N., Birolo G., De Pascale F., Feltrin E., Schiavon R., Anglani F., Negrisolo S., Zanetti A. et al. (2017). QueryOR: a comprehensive web platform for genetic variant analysis and prioritization. *BMC Bioinformatics* 18, 225 10.1186/s12859-017-1654-4 28454514 PMC5410040

[DMM032839C3] Blake J. A., Eppig J. T., Kadin J. A., Richardson J. E., Smith C. L., Bult C. J. and the Mouse Genome Database Group (2017). Mouse Genome Database (MGD)-2017: community knowledge resource for the laboratory mouse. *Nucleic Acids Res.* 45, D723-D729. 10.1093/nar/gkw1040 27899570 PMC5210536

[DMM032839C4] Campbell M. P. and Packer N. H. (2016). UniCarbKB: new database features for integrating glycan structure abundance, compositional glycoproteomics data, and disease associations. *Biochim. Biophys. Acta Gen. Subj.* 1860, 1669-1675. 10.1016/j.bbagen.2016.02.016 26940363

[DMM032839C5] Cheng L., Jiang Y., Wang Z., Shi H., Sun J., Yang H., Zhang S., Hu Y. and Zhou M. (2016). DisSim: an online system for exploring significant similar diseases and exhibiting potential therapeutic drugs. *Sci. Rep.* 6, e99415 10.1038/srep30024 PMC4960572 27457921

[DMM032839C6] Davis A. P., Wiegers T. C., Rosenstein M. C. and Mattingly C. J. (2012). MEDIC: a practical disease vocabulary used at the Comparative Toxicogenomics Database. *Database* 2012, bar065 10.1093/database/bar065 22434833 PMC3308155

[DMM032839C7] Eppig J. T., Smith C. L., Blake J. A., Ringwald M., Kadin J. A., Richardson J. E. and Bult C. J. (2017). Mouse Genome Informatics (MGI): resources for mining mouse genetic, genomic, and biological data in support of primary and translational research. *Methods Mol. Biol.* 1488, 47-73. 10.1007/978-1-4939-6427-7_3 27933520

[DMM032839C8] Farlow J., Pankratz N. D., Wojcieszek J. and Foroud T. (2004). Parkinson Disease Overview. In *GeneReviews®* (ed. M. P. Adam, H. H. Ardinger, R. A. Pagon, S. E. Wallace, L. J. H. Bean, K. A. A. Stephens). Seattle: University of Washington.

[DMM032839C9] Fernandez-Llimos F., Minguet F. and Salgado T. M. (2017). New pharmacy-specific Medical Subject Headings included in the 2017 database. *Am. J. Heal. Pharm.* 74, 1128-1129. 10.2146/ajhp170046 28743776

[DMM032839C10] Griffith M., Spies N. C., Krysiak K., McMichael J. F., Coffman A. C., Danos A. M., Ainscough B. J., Ramirez C. A., Rieke D. T., Kujan L. et al. (2017). CIViC is a community knowledgebase for expert crowdsourcing the clinical interpretation of variants in cancer. *Nat. Genet.* 49, 170-174. 10.1038/ng.3774 28138153 PMC5367263

[DMM032839C11] Hayman G. T., Laulederkind S. J. F., Smith J. R., Wang S.-J., Petri V., Nigam R., Tutaj M., De Pons J., Dwinell M. R. and Shimoyama M. (2016). The Disease Portals, disease–gene annotation and the RGD disease ontology at the Rat Genome Database. *Database* 2016, baw034 10.1093/database/baw034 27009807 PMC4805243

[DMM032839C12] Hoehndorf R., Gruenberger M., Gkoutos G. V. and Schofield P. N. (2015). Similarity-based search of model organism, disease and drug effect phenotypes. *J. Biomed. Semantics* 6, 6 10.1186/s13326-015-0001-9 25763178 PMC4355138

[DMM032839C26] Kibbe W. A., Arze C., Felix V., Mitraka E., Bolton E., Fu G., Mungall C. J., Binder J. X., Malone J., Vasant D. et al. (2015). Disease Ontology 2015 update: an expanded and updated database of human diseases for linking biomedical knowledge through disease data. *Nucleic Acids Res.* 43, D1071-D1078. 10.1093/nar/gku1011 25348409 PMC4383880

[DMM032839C13] Köhler S., Vasilevsky N. A., Engelstad M., Foster E., McMurry J., Aymé S., Baynam G., Bello S. M., Boerkoel C. F., Boycott K. M. et al. (2017). The human phenotype ontology in 2017. *Nucleic Acids Res.* 45, D865-D876. 10.1093/nar/gkw1039 27899602 PMC5210535

[DMM032839C14] Kozaki K., Yamagata Y., Mizoguchi R., Imai T. and Ohe K. (2017). Disease Compass– a navigation system for disease knowledge based on ontology and linked data techniques. *J. Biomed. Semantics* 8, 22 10.1186/s13326-017-0132-2 28629436 PMC5477351

[DMM032839C15] Motenko H., Neuhauser S. B., O'Keefe M. and Richardson J. E. (2015). MouseMine: a new data warehouse for MGI. *Mamm. Genome* 26, 325-330. 10.1007/s00335-015-9573-z 26092688 PMC4534495

[DMM032839C16] Mungall C. J., Torniai C., Gkoutos G. V., Lewis S. E. and Haendel M. A. (2012). Uberon, an integrative multi-species anatomy ontology. *Genome Biol.* 13, R5 10.1186/gb-2012-13-1-r5 22293552 PMC3334586

[DMM032839C17] Orechia J., Pathak A., Shi Y., Nawani A., Belozerov A., Fontes C., Lakhiani C., Jawale C., Patel C., Quinn D. et al. (2015). OncDRS: an integrative clinical and genomic data platform for enabling translational research and precision medicine. *Appl. Transl. Genomics* 6, 18-25. 10.1016/j.atg.2015.08.005 PMC4803771 27054074

[DMM032839C18] Patterson S. E., Liu R., Statz C. M., Durkin D., Lakshminarayana A. and Mockus S. M. (2016). The clinical trial landscape in oncology and connectivity of somatic mutational profiles to targeted therapies. *Hum. Genomics* 10, 4 10.1186/s40246-016-0061-7 26772741 PMC4715272

[DMM032839C19] Rath A., Olry A., Dhombres F., Brandt M. M., Urbero B. and Ayme S. (2012). Representation of rare diseases in health information systems: the orphanet approach to serve a wide range of end users. *Hum. Mutat.* 33, 803-808. 10.1002/humu.22078 22422702

[DMM032839C20] Schriml L. M. and Mitraka E. (2015). The Disease Ontology: fostering interoperability between biological and clinical human disease-related data. *Mamm. Genome* 26, 584-589. 10.1007/s00335-015-9576-9 26093607 PMC4602048

[DMM032839C21] Schriml L. M., Arze C., Nadendla S., Chang Y.-W. W., Mazaitis M., Felix V., Feng G. and Kibbe W. A. (2012). Disease Ontology: a backbone for disease semantic integration. *Nucleic Acids Res.* 40, D940-D946. 10.1093/nar/gkr972 22080554 PMC3245088

[DMM032839C22] Smith B., Ceusters W., Klagges B., Köhler J., Kumar A., Lomax J., Mungall C., Neuhaus F., Rector A. L. and Rosse C. (2005). Relations in biomedical ontologies. *Genome Biol.* 6, R46 10.1186/gb-2005-6-5-r46 15892874 PMC1175958

[DMM032839C23] Smith B., Ashburner M., Rosse C., Bard J., Bug W., Ceusters W., Goldberg L. J., Eilbeck K., Ireland A., Mungall C. J. et al. (2007). The OBO Foundry: coordinated evolution of ontologies to support biomedical data integration. *Nat. Biotechnol.* 25, 1251-1255. 10.1038/nbt1346 17989687 PMC2814061

[DMM032839C24] Vitali F., Cohen L. D., Demartini A., Amato A., Eterno V., Zambelli A. and Bellazzi R. (2016). A network-based data integration approach to support drug repurposing and multi-target therapies in triple negative breast cancer. *PLoS ONE* 11, e0162407 10.1371/journal.pone.0162407 27632168 PMC5025072

